# Global Transcriptome Analysis of Orange Wheat Blossom Midge, *Sitodiplosis mosellana* (Gehin) (Diptera: Cecidomyiidae) to Identify Candidate Transcripts Regulating Diapause

**DOI:** 10.1371/journal.pone.0071564

**Published:** 2013-08-05

**Authors:** Zhong-Jun Gong, Yu-Qing Wu, Jin Miao, Yun Duan, Yue-Li Jiang, Tong Li

**Affiliations:** Institute of Plant Protection, Henan Academy of Agricultural Sciences, Key Laboratory of Crop Pest Control of Henan Province, Key Laboratory of Crop Integrated Pest Management of the Southern of North China, Ministry of Agriculture of the People’s Republic of China, Zhengzhou, China; Auburn University, United States of America

## Abstract

**Background:**

Many insects enter a developmental arrest (diapause) that allows them to survive harsh seasonal conditions. Despite the well-established ecological significance of diapause, the molecular basis of this crucial adaptation remains largely unresolved. *Sitodiplosis mosellana* (Gehin), the orange wheat blossom midge (OWBM), causes serious damage to wheat throughout the northern hemisphere, and sporadic outbreaks occur in the world. Traits related to diapause appear to be important factors contributing to their rapid spread and outbreak. To better understand the diapause mechanisms of OWBM, we sequenced the transcriptome and determined the gene expression profile of this species.

**Methodology/Principal Findings:**

In this study, we performed de novo transcriptome analysis using short-read sequencing technology (Illumina) and gene expression analysis with a tag-based digital gene expression (DGE) system. The sequencing results generated 89,117 contigs, and 45,713 unigenes. These unigenes were annotated by Blastx alignment against the NCBI non-redundant (nr), Clusters of orthologous groups (COG), gene orthology (GO), and the Kyoto Encyclopedia of Genes and Genomes (KEGG) databases. 20,802 unigenes (45.5% of the total) matched with protein in the NCBI nr database. Two digital gene expression (DGE) libraries were constructed to determine differences in gene expression profiles during diapause and non-diapause developmental stages. Genes related to diapause were analyzed in detail and in addition, nine diapause-related genes were analyzed by real time PCR.

**Conclusions/Significance:**

The OWBM transcriptome greatly improves our genetic understanding and provides a platform for functional genomics research of this species. The DGE profiling data provides comprehensive information at the transcriptional level that facilitates our understanding of the molecular mechanisms of various physiological aspects including development and diapause stages in OWBM. From this study it is evident that various genes coding metabolic enzymes are crucial for diapause and metamorphosis.

## Introduction

*Sitodiplosis mosellana* (Gehin), the orange wheat blossom midge (OWBM), causes serious damage to wheat (Triticum aestivum L.) throughout the northern hemisphere, and sporadic outbreaks occur in North America, Europe and Asia [1], [2]. In northern China, two major outbreaks of wheat midge were recorded in the 1950s and 1980s, causing serious crop loss during the years [3]. Since 2000, wheat midge populations have been observed in northern China every year largely due to changes in weather conditions and crops distribution [4]. Wheat midge development consists of one generation per year with mature larvae falling from wheat ears to the ground from mid to late May. And most wheat midge larvae burrow 6–10 cm into the soil before forming a protective round cocoon. Once in the soil, wheat midge larvae can successfully diapause, in which they over-summer and over-autumn during obligatory diapause. Diapause occurs at the mature third instar larvae in each generation, regardless of the prevailing environmental conditions [5]. With decreasing ambient temperatures during winter, this diapause is gradually being terminated leaving larvae in an inactive state of post-diapause quiescence. Most studies on the diapause of wheat blossom midge have focused on the ecology and biochemistry [6]–[10] with a very few studies on the molecular mechanisms that control diapause [11].

Temperature and soil moisture have been shown to affect the development of OWBM [12]. At low temperatures wheat midge larvae do not pupate. Therefore, a long diapause is important to maintain synchrony between the life cycle of OWBM and the phenology of wheat. Diapause is an alternate life-history trait that enables individuals to endure unfavorable environmental conditions such as drought and cold. During diapause, which is a dormancy common to over-wintering insects, development is usually arrested by suppressed/blocked metabolic pathways, and a unique pattern of gene expression is evoked [13]. After termination of diapause, metabolic rate rapidly increases to initiate development.

Currently, insect diapause is considered as a dynamic (rather than static) process consisting of several successive phases [14]. However, the mechanisms that maintain and terminate diapause are poorly understood. Diapause and non-diapause phases of an insect's life cycle are strikingly contrast and the underlying differences at the molecular level remain largely unknown [15].

Only a few genes expressed in the OWBM have been characterized thus far, including the serine carboxypeptidase and serine proteases, and genes involved in diapause have not been studied in this insect. A global analysis of the OWBM transcriptome should, therefore, provide a comprehensive information on the physiological and biochemical processes of this insect species. In this study, we generated over 4.5 billion bases of high-quality DNA sequences using Illumina sequencing technology and demonstrated the suitability of short-read sequencing for de novo assembly and annotation of genes expressed in an eukaryote without prior genome information. In total, we obtained 45,713 unigenes from the OWBM transcriptome. Further, we also compared the gene expression profiles of diapause OWBM larvae (DOL) and non-diapause OWBM larvae (NDOL) using a digital gene expression (DGE) system. The assembled, annotated transcriptome sequences and gene expression profiles will be a valuable resource to identify OWBM genes involved in development modulation and diapause.

## Methods

### Ethics Statement

The cocoons of OWBM used in this study were originally collected from Wuzhi, Henan province, China. The field studies did not involve endangered or protected species. No specific permits were required for the described field collections, and the location is not protected in any way. OWBM is common agricultural pest and is not included in the “List of Protected Animals in China”.

### Insects

The cocoons of OWBM were originally collected during March 2011 and reared at 20°C and 70±10% relative humidity. The third instar larvae, pupae and adults were collected, respectively. Finally, these samples were frozen at −80°C until use.

For the DGE experiments, NDOL were collected from fields in late May in Wuzhi, and reared under 4°C condition for three months in laboratory to break diapause. DOL were directly collected from the same locality in August. Before collection, all freshly samples were transported to the laboratory and maintained three days at 20°C and 16∶8 h light:dark photoperiod. Both DOL and NDOL samples were frozen in liquid nitrogen and stored at −80°C until further analysis.

### RNA Isolation and Library Preparation for Transcriptome Analysis

Total RNA was extracted using standard protocols (Trizol; Invitrogen, Carlsbad, CA, USA) after powdering the tissues in liquid nitrogen. Pooled RNA sample from different developmental stages (cocoon, third instar larvae, pupae and adults) were used for transcriptome analysis. RNA integrity was confirmed using the 2100 Bioanalyzer (Agilent Technologies) with a minimum RNA integration value of 6. Samples for transcriptome analysis were prepared using the ScriptSeq™ mRNA-seq Library Preparation Kit (Illumina, San Diego, CA USA) following the manufacturer’s recommendations. Briefly, mRNA was purified from 6 µg total RNA using oligo(dT) magnetic beads. Following purification, the mRNA was fragmented into small pieces using divalent cations under elevated temperatures and the cleaved RNA fragments were then used for first strand cDNA synthesis using reverse transcriptase and random hexamer-primers. This was followed by second-strand cDNA synthesis using buffer, dNTPs, DNA polymerase I and RNaseH. Short fragments were purified with a QiaQuick PCR extraction kit (Qiagen). These fragments were washed with EB buffer for end repair and adding poly (A), and then ligated to sequencing adapters. Approximately 200 bp fragments, as judged by agarose gel electrophoresis, were selected as templates for PCR amplification. The cDNA library was sequenced on Illumina HiSeq™ 2000 using paired-end technology in a single run. The data sets are available at the NCBI Short Read Archieve (SRA) with the accession number: SRX252524.

### Analysis of Illumina Sequencing Results

De novo assembly of the transcriptome was carried out with the short reads assembling program – Trinity [16]. Reads with certain length of overlap were combined to form longer fragments, called contigs. Then the reads were mapped back to contigs to detect contigs from the same transcript as well as the distances between these contigs. The contigs were then connected to obtain sequences that could not be extended on either ends. Such sequences were defined as unigenes. In the final step, Blastx comparisons (E-value <10–5) were performed between unigenes and proteins in databases such as NCBI nr, Swiss-Prot, KEGG and COG, and the best results were used to decide sequence direction of unigenes. If conflicting results were obtained from different databases priority was given in the order of NCBI nr, Swiss-Prot, KEGG and COG before deciding sequence direction of unigenes. When a unigene did not align to any protein in the above databases, the ESTScan software [17] was used to decide its sequence direction. This Transcriptome Shotgun Assembly project has been deposited at DDBJ/EMBL/GenBank under the accession GAKJ00000000. The version described in this paper is the first version, GAKJ01000000.

### Digital Gene Expression Library Preparation and Sequencing

Total RNA was extracted separately from DOL and NDOL using Trizol Reagent following manufacturer’s instructions. Approximately 10 µg RNA from each sample was used to construct individual DGE libraries. mRNA was isolated as described under cDNA library construction. The cDNA fragments were purified by agarose gel electrophoresis and enriched by PCR amplification. Each library had an insert size of 200 bp, and 42–50 bp sequences were generated by Illumina HiSeq 2000 in a single run using paired-end technology. The data discussed in this publication have been deposited in NCBI's Gene Expression Omnibus and are accessible through GEO Series accession number GSE48156 (http://www.ncbi.nlm.nih.gov/geo/query/acc.cgi? acc = GSE48156).

### Analysis and Mapping of DGE Tags

The original image data were transferred into sequence data by base calling and was defined as raw data. To obtain clean reads, all dirty raw reads were removed before data analysis. Dirty raw reads were defined as (1) reads with adaptors, (2) reads with more than 10% unknown bases and (3) low quality reads with more than 50% low quality bases with quality value ≤5. Clean reads were mapped to reference sequences (unigenes from transcriptome data of different developmental stages) using SOAPaligner/soap2 [18]. No more than two base mismatches were allowed in the alignment. The gene expression level was calculated using the RPKM method (Reads Per kb per Million reads) [19]. If there were more than one transcript for a gene, the longest one was then used to calculate its expression level and coverage. To identify differentially expressed genes between two samples, the false discovery rate (FDR) method was used to determine the threshold of P-value in multiple tests [20]. The significance of gene expression difference was judged using a threshold FDR ≤0.001 and an absolute value of log2 Ratio ≥1. The genes that were expressed at different levels across samples were further annotated by GO functional analysis and KEGG pathway analysis.

### Quantitative Real-time PCR (qRT-PCR) Validation

Quantitative RT-PCR technique was used to verify deep sequencing results of DOL and NDOL samples. In addition, total RNA of OWBM at different development stages was extracted separately, and 1 µg total RNA from each sample was reverse-transcribed in a 20 µl reaction to generate the first-strand cDNA using the PrimeScript RT reagent Kit with gDNA Eraser (TaKaRa). Development stages included the cocoon, larvae, pre-pupae, early-pupae, mid-pupae, post-pupae, adult females and adult males. All primers designed for real-time PCR analyses are listed in Table S9 in File S1. Real-time PCR was carried out using SYBR Premix Ex Taq II (Tli RNaseH Plus) (TaKaRa) according to the manufacturer’s instructions in the StepOne Plus Real-time PCR system (Applied Biosystems) using the following cycling conditions: 30 s at 95°C followed by 40 cycles of 95°C for 5s and 60°C for 30 s. Specificity of the primers was confirmed by reverse transcription PCR (RT-PCR) (data not shown) and melting curve analysis. All reactions were carried out in triplicate, and the average C_t_ value was used to quantify the relative copy number. All results were normalized against the expression level of the OWBM 18srRNA gene. Relative expression levels across samples were measured based on the 2^−ΔΔCt^ method [21].

## Results

### Sequencing and Assembly

**Figure 1:**
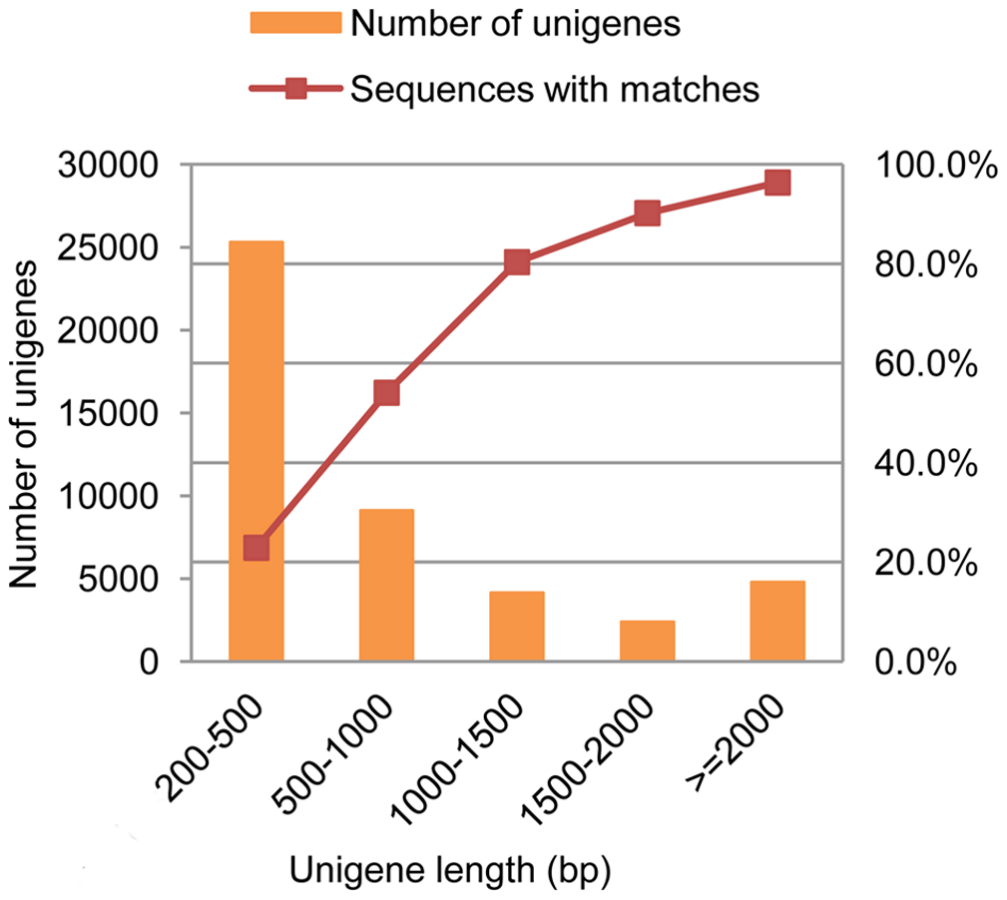
Unigene size distribution and proportion of sequences with matches. All unigenes sizes were calculated.

To obtain a global transcriptome of the entire OWBM life cycle, a cDNA sample was prepared from larvae, pupae and adults followed by high throughput RNA-seq. A total of 51,212,118 clean reads (accumulated length of 4,609,090,620 bp) were generated by the Illumina HiSeq™ 2000 sequencing. These reads were assembled into 89,117 contigs with a mean length of 421 bp and N50 of 909 bp (i.e. 50% of the total assembled sequence was present in contigs of this length or longer). After connecting the contigs using Trinity, we obtained 45,713 unigenes with a mean length of 854 bp and N50 of 1,538 bp, including 487 clusters and 45, 226 singletons. The size distribution indicated that the lengths of the 11,304 unigenes were more than 1000 bp (24.73% of all unigenes) (Figure 1).

### Annotation of Predicted Proteins

**Figure 2:**
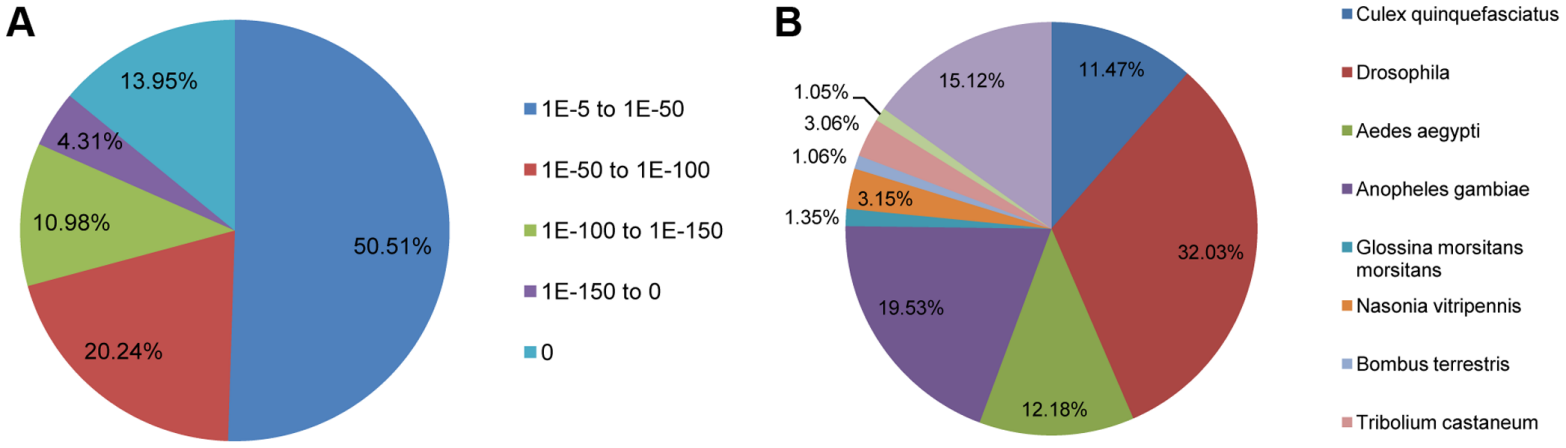
Analysis of Illumina sequences in the NCBI nr database. A. E-value distribution; B. Species distribution.

To annotate the unigenes, we first compared our OWBM reference sequences against the NCBI non-redundant (nr) protein database using Blastx with a cut-off E-value of 10^−5^. A total of 20,802 (45.5% of all distinct sequences) unigenes resulted from the search (Table S1 in File S1). Figure 1 indicates that the proportion of sequences with matches in nr databases is greater among the longer unigenes. More than 80% match efficiency was observed for sequences longer than 1, 000 bp while the match efficiency was only 22.9% for those ranging from 200 to 500 bp. The E-value distribution of the top hits with 50.5% of homologous unigenes ranged between 1.0E-5 to 1.0E-50, whereas 49.5% had strong homology (smaller than 1.0E-50) (Figure 2A).

Species distribution of the best match for each sequence is shown in Figure 2B. The *S. mosellana* sequences showed 32.03% matches with Drosophila spp sequences followed by *Anopheles gambiae* (19.53%), *Aedes aegypti* (12.18%), and *Culex quinquefasciatus* (11.47%).

### Gene Ontology (GO), Clusters of Orthologous Groups (COG) and Kyoto Encyclopedia of Genes and Genomes (KEGG) Ontology (KO) Classification

**Figure 3:**
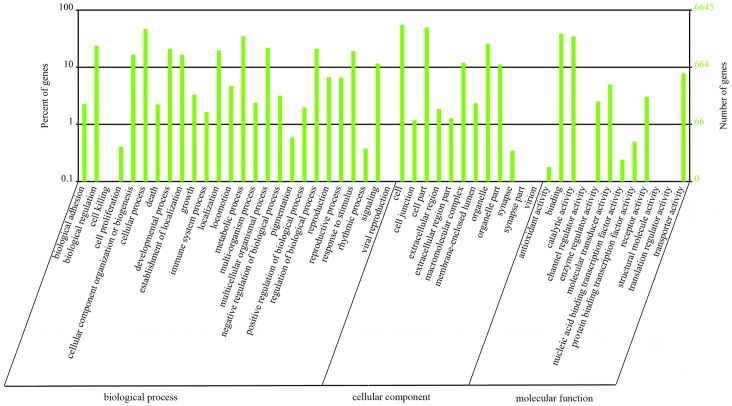
GO categories of the unigenes. Unigenes were annotated into three categories: biological process, cellular component and molecular function.

We used GO and KO assignments to classify functions for the predicted *S. mosellana* unigenes. Among the 20,802 unigenes, approximately 31.94% could be annotated in GO, and 72.18% could be annotated in KO based on sequence homology (Table S2 and Table S3 in File S1). Among the three main categories (biological process, cellular component and molecular function) of the GO classification, ‘cellular process’, ‘cell and cell part’, and ‘binding and catalytic activity’ terms were predominant in each category, respectively (Figure 3).

**Figure 4:**
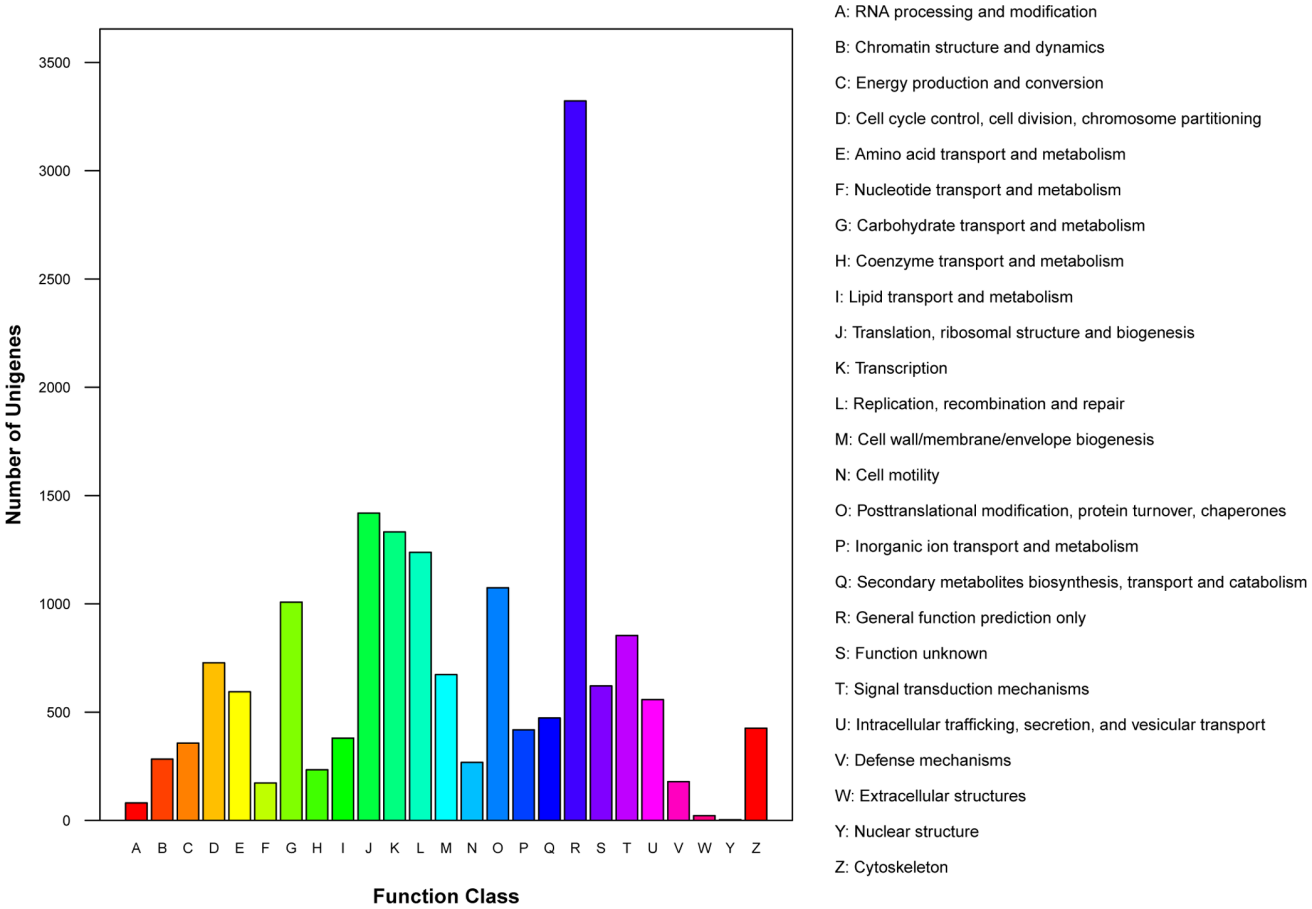
Classification of COG for the *Sitodiplosis mosellana* transcriptome. 7,974 unigenes (34.96% of the total) were annotated and divided into 25 specific categories.

To further evaluate our transcriptome library and the effectiveness of our annotation process, we searched the annotated sequences for the genes involved in COG classifications. From a total of 20,802 nr hits 7,974 sequences had a COG classification (Figure 4). Among the 25 COG categories, the cluster for ‘general function prediction’ represented the largest group (3322, 19.87%) followed by ‘translation, ribosomal structure and biogenesis’ (1419, 8.49%), ‘transcription’ (1332, 7.97%) and ‘replication, recombination and repair’ (1238, 7.41%). The following categories: RNA processing and modification (81, 0.48%), extracellular structures (22, 0.13%) and nuclear structure (3, 0.02%), represented the smallest groups (Figure 4).

In total 15,014 unigenes were annotated to KEGG. About 242 pathways were obtained including ‘insulin signaling pathway’ (263 members), ‘steroid hormone biosynthesis’ (91 members), ‘TGF-beta signaling pathway’ (114 members) and ‘endocrine and other factor-regulated calcium reabsorption’ (74 members).

### Digital Gene Expression (DGE) Library Sequencing

We sequenced two DGE libraries: NDOL which experienced a low temperature and DOL which was not exposed to low temperature. The two libraries generated 7.39 and 7.29 million raw tags, respectively. After removing low quality reads, the total number of tags in NDOL and DOL were 7.36 and 7.27 million, respectively. The number of distinctly aligned tags was similar between each library. Among these clean reads, 6 million (81.5%) and 5.4 million (73.8%), were mapped to OWBM unigenes. The percentage of clean tags among the raw tags in NDOL and DOL were 99.67 and 99.66%, reflecting the high quality of sequencing (Figure S1 A in File S1). To evaluate the DGE data, we analyzed the distribution of the expression of all unique reads (Figure S1 B in File S1). Among the unique reads in each library, percentages of the highly expressed genes, i.e., genes with more than 100 copy numbers, were greater than 13%. In contrast, genes with a low level of expression, i.e., genes with less than five copy numbers, showed a broad distribution of distinct clean tags in each library (above 31%) and their distribution was also consistent between the two libraries.

To confirm whether the number of detected genes increased proportional to the numbers sequenced (total tag number), a saturation analysis was performed. There was an initial steep increase in the number of *S. mosellana* transcripts discovered with increase in read numbers. Slope of the curve showed a saturation trend where the number of detected genes almost ceased to increase. The numbers of reads at this time reached 6 million (Figure S2 in File S1). In this study, the total number of tags in each library was above 7.29 million, implying that our sequencing depth captured most of the *S. mosellana* transcripts.

About 19.4 and 14.9% of genes had 90–100% coverage in NDOL and DOL, respectively. Fewer than 5.1% of the genes had 0–10% coverage and the distribution of genes with more than 50% coverage was 64.0% in NDOL and 57.1% in DOL (Figure S1 C in File S1).

### Comparison of Gene Expression Profile among the Different Development Stages

**Figure 5:**
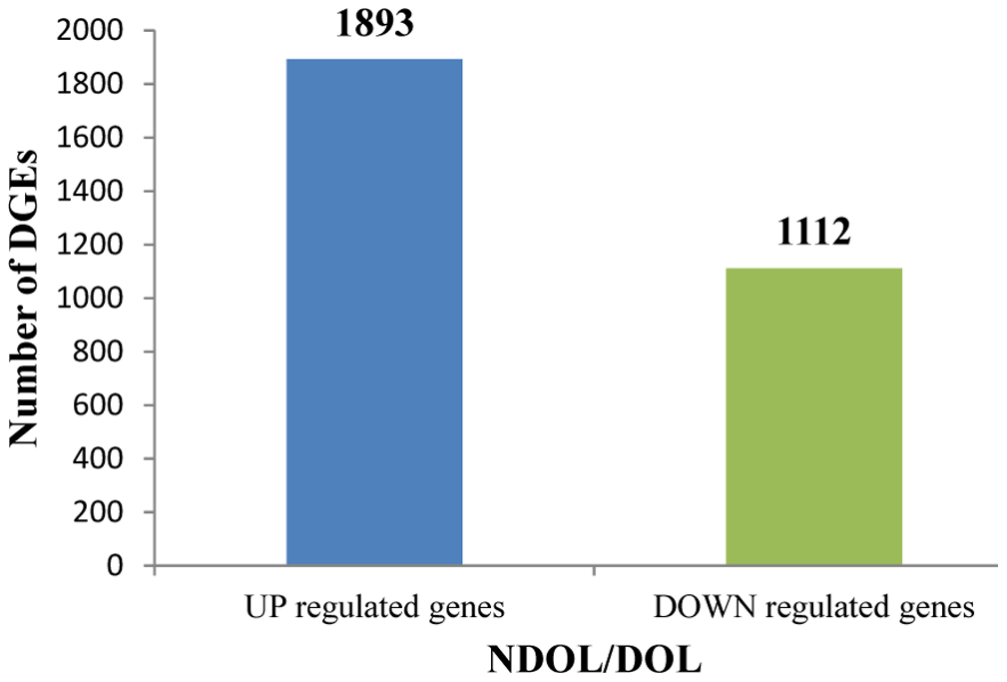
Differences in gene expression profile between the DOL and NDOL. ‘DGEs’ indicate unigenes with FDR ≤0.001 and an absolute value of log2Ratio ≥1. Numbers of genes up-regulated and down-regulated in the NDOL compared to DOL are indicated above the blue or green bars, respectively.

Based on the threshold level of significant differences in gene expression, 25,404 unigenes were up-regulated and 14,843 were down-regulated. Further, significance of the differences in gene expression was judged by the threshold of false discovery rate (FDR ≤0.001) and the absolute value of log2 Ratio (≥1). The results revealed 3,005 genes with significant differential expression between NDOL and DOL, with 1,893 up-regulated and 1,112 genes down-regulated in NDOL compared to DOL (Figure 5, Table S4 in File S1).

Of the ten most differentially up-regulated genes only three had defined functions that matched the genes encoding guanylyl cyclase receptor of *C. quinquefasciatus* (XP_001845718), disulfide isomerase of *C. quinquefasciatus* (XP_001847538), and cytochrome P450 of *A. aegypti* (XP_001659149). One gene was homologous to a *Nasonia vitripennis* gene (XP_001602000) of unknown function (Table S5 in File S1). Six other genes also had no known functions or annotations.

One of the ten most differentially down-regulated genes had defined functions that matched the gene encoding hexamerin 2 beta and six other genes showed homology with genes in the NCBI nr database. Three genes could not be annotated (Table S5 in File S1).

According to the GO classification, most of the gene sets demonstrated up-regulated expression in the NDOL library, and these genes correlated to metabolic process and catabolic process (Table S6 in File S1). Considering the Molecular Function category, most genes were addressed to hydrolase activity (181/64 up- and down-regulation comparing DNOL and DOL, respecitvely) and nucleotide binding (105/68 up- and down-regulation comparing DNOL and DOL, respectively). The groups with much fewer terms (only 6 protein annotated) include mannosidase activity and O-acyltransferase activity.

To explore the biological function of the significantly differentially expressed genes, all 3,005 genes were mapped to the KEGG database. These were involved in important pathways such as proteasome, ribosome and protein processing in the endoplasmic reticulum. There were also 1,300 unigenes mapped to 206 pathways. In the KO classification, 12 gene sets were significantly enriched, and the expressions of 255 and 189 genes were found up- and down-regulated, respectively. In the proteasome pathway category, 35 genes were significantly up-regulated and only 2 were down-regulated. In the cell cycle pathway category, 32 genes were significantly up-regulated and only 6 were down-regulated. In addition, our results indicated that all of the genes (63 genes) involved in ribosome pathway were down-regulated (Table S6 in File S1).

### Heat Shock Protein (Hsp) Expression

**Figure 6:**
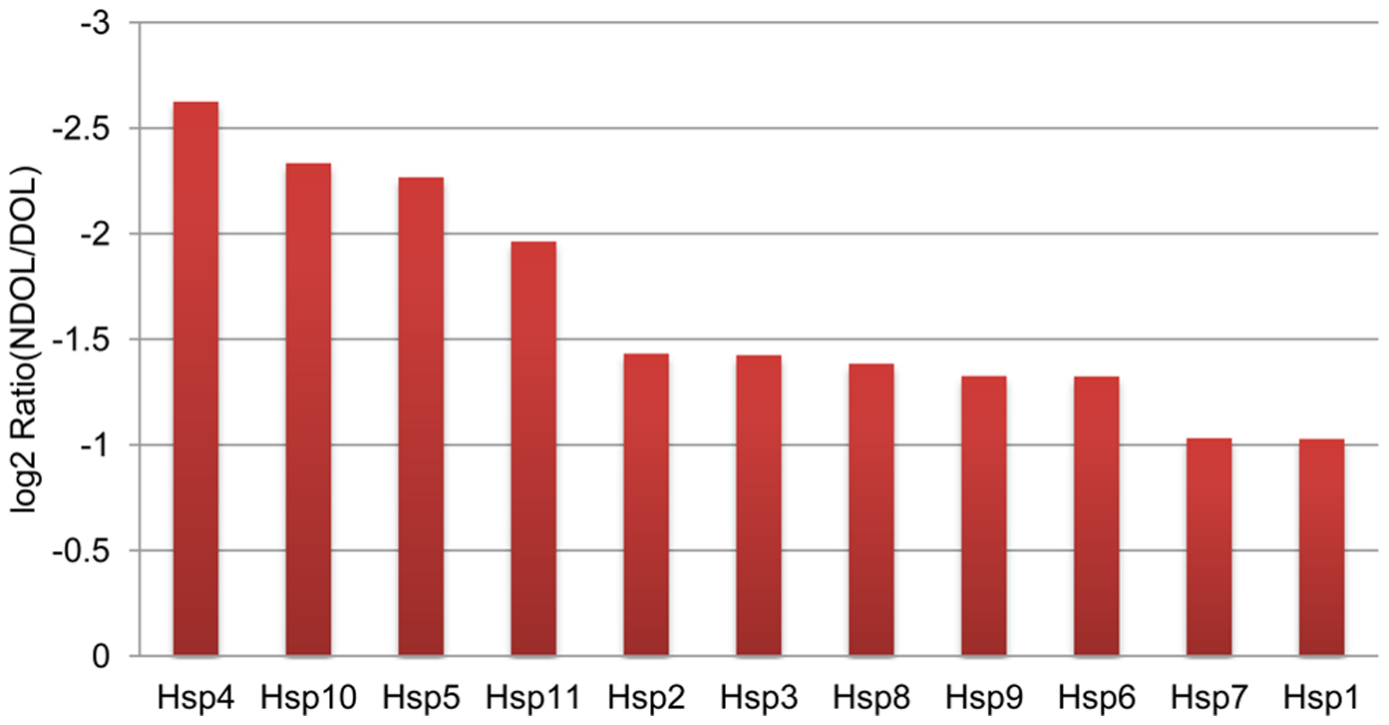
Expression profiling of the *S. mosellana* HSPs genes in DOL and NDOL.

The up-regulation of Hsps appears to be common to diapausing dipterans. We identified 43 Hsp unigenes in the *S. mosellana* transcriptome. Among them, five were similar to small heat shock proteins (sHsps) and four sHsps (Unigene11947, Unigene17774, Unigene18840, Unigene19156) appeared to be complete sequences. Only eleven unigenes (Hsp1–11) with significant differences in gene expression between NDOL and DOL were identified and all were down-regulated in NDOL compared to DOL (Figure 6, Table S7 in File S1).

### Metabolic Enzyme Gene Expression

**Figure 7:**
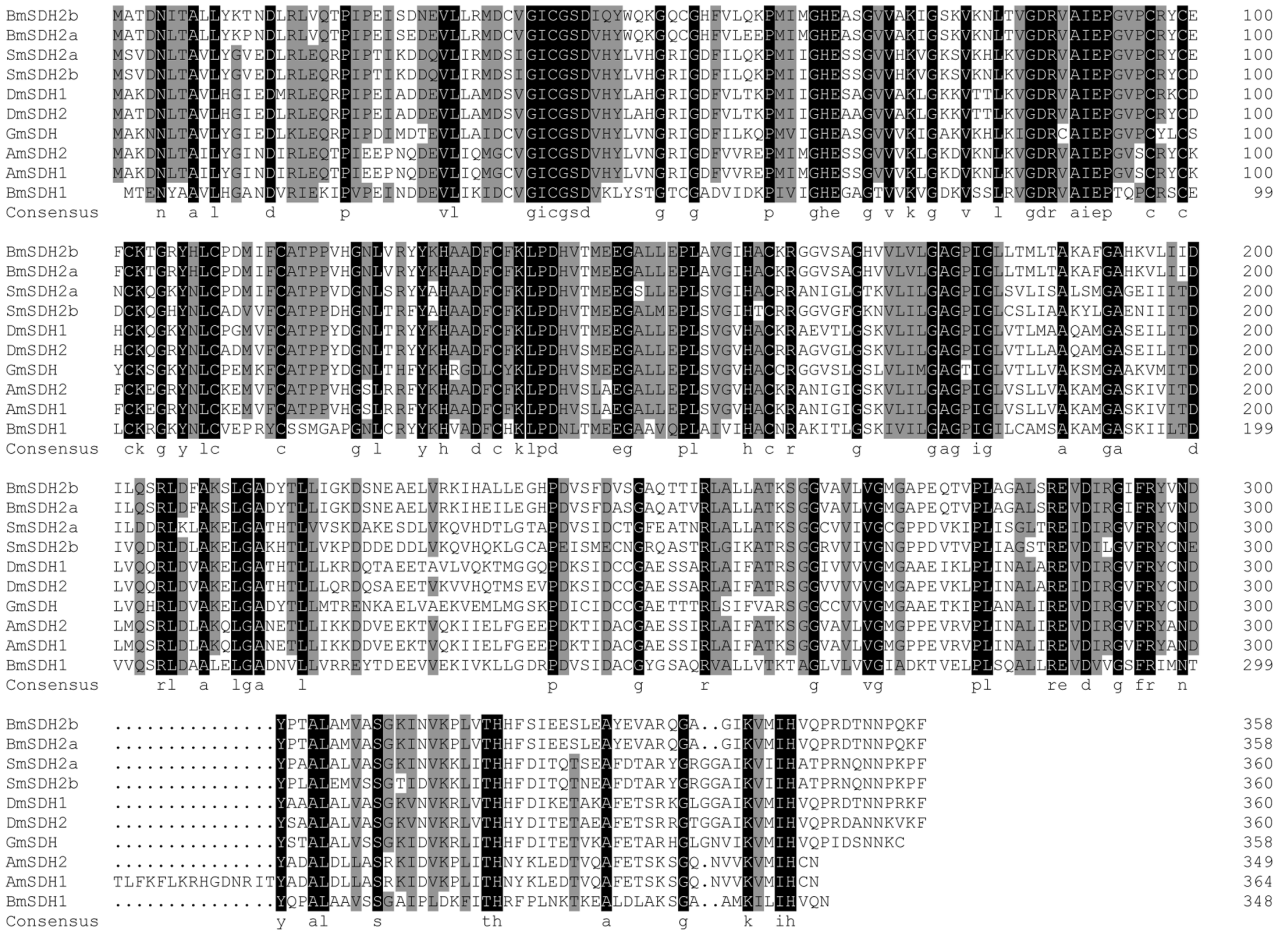
Sequence alignment of SDH genes. GenBank accession numbers of the sequences are: *Apis mellifera*, AmSDH1 (XP_003251602) and AmSDH2 (XP-392401), *Drosophila melanogaster*, DmSDH1 (NP_477348) and DmSDH2 (NP_524311), *Glossina morsitans morsitans*, GmSDH (ADD19424), *Bombyx mori*, BmSDH1 (D13371), BmSDH2a (AB164059) and BmSDH2b (AB604175).

We then focused on the genes related to diapause. In OWBM, the relative levels of transcripts of two genes coding for NAD-sorbitol dehydrogenase (SDH)2 (SDH2a and SDH2b), which are important in carbohydrate metabolism coverting sorbitol into fructose, were significantly lower in NDOL than DOL. Sequence alignments and phylogenetic analysis showed that SDH genes of OWBM shared high sequence identity with homologs from other dipetrans (Figure 7, Figure S3 in File S1). There were two unigenes (Unigene6351 and Unigene7576) encoding proteins (TREH1 and TREH2) with trehalase activity, which are important in the conversion of trehalose to glucose, also had differential expression pattern with the expression of TREH1 up-regulated in NDOL than DOL and expression of TREH2 down-regulated in NDOL than DOL. In addition, Glycogen phosphorylase (GP), which is important in glycogenolysis, was significantly lower in NDOL than DOL and fructose-bisphosphate aldolase (FBPA) was also down-regulated in NDOL than DOL. FBPA is an early step in the glycolysis pathway and the products of this pathway are ATP and pyruvic acid (PVA).

Juvenile hormone epoxide hydrolase (JHEHs) and juvenile hormone esterase (JHE) which were down-regulated in NDOL than DOL, both lead to suppression of JH signaling and response. Additionally, isocitrate dehydrogenase (IDH), which is important component of the tricarboxylic acid cycle (TCA cycle), was up-regulated in NDOL than DOL.

### qRT-PCR Analysis

**Figure 8:**
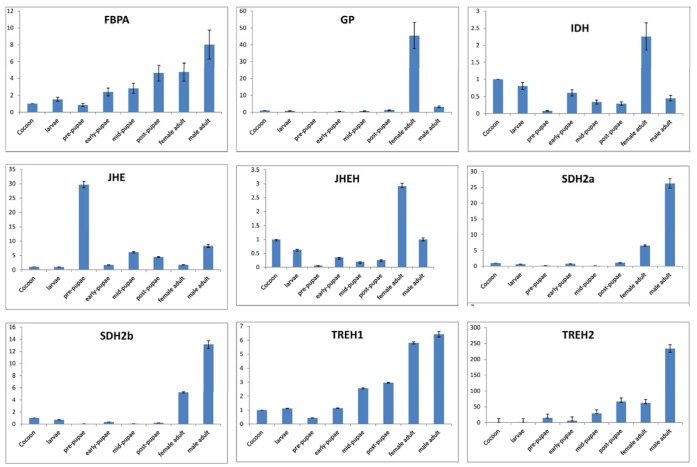
Expression profiling of the *S. mosellana* metabolic enzyme genes in different developmental stages.

To validate our deep sequencing data, nine differentially regulated *S. mosellana* genes were selected from diapause-related genes for qRT-PCR analysis. They were FBPA, GP, IDH, JHE, JHEH, SDH2a, SDH2b, TREH1, TREH2. Except for one of the genes (JHE), the qRT-PCR results correlated with the data obtained from deep sequencing analysis showing similar trends in up- or down-regulation of OWBM genes. For example, based on deep sequencing analysis, FBPA, TREH2 were down-regulated 1.23 and 0.52 fold, respectively, and showed 1.18 and 0.75 fold changes, respectively in qRT-PCT analyses (Table S8 in File S1). This indicated that the results of change detected by DGE analysis are reliable. To determine the involvement of genes encoding metabolic enzymes in OWBM development, we further analyzed these associated genes (Figure 8). All nine genes had similar expression in the cocoon and larval stages. Compared to cocoon, the expression level of *JHE* increased significantly in pre-larvae and reduced to a lower level in early-pupae. *SDH2a, SDH2b* and *GP* had lower expression levels in larval and pupal stages but higher levels in adults. While *SDH* genes were highly expressed in adult males, *GP* was highly expressed in adult females. *FBPA* and *TREH1* were down-regulated at the stage of pre-pupae compared to the former stages and then the expression level of the two genes increased gradually from early-pupae to adult. *TREH2* were up-regulated in pre-pure and then down-regulated in early-pupae. *IDH* and *JHEH* had similar expression patterns and the expression levels of both genes decreased in pre-pupae.

## Discussion

The orange wheat blossom midge, *S. mosellana* (Gehin), is a serious pest to wheat. Although the ecological and physiological mechanisms associated with OWBM have been studied extensively, the lack of genetic information and molecular mechanisms related to diapause is a barrier to further understand this species. Our preliminary study indicated that larvae not exposed to low temperatures remained in diapause state and did not pupate (unpublished data, Gong *et al*., 2013). Diapause traits appear to be important factors that contribute to the rapid spread and outbreak of OWBM.

Based on the transcriptome analysis, we detected 45,713 unigenes in OWBM. Using Blastx 45.5% of them (20,802) were found to have significant homology to genes encoding functional proteins. Among them, 11,304 unigenes were more than 1,000 bp (24.73% of all unigenes) indicating that the sequence quality was high enough for further analysis. Homology analysis of the unigenes demonstrated that 32.03% were similar to *Drosophila*, and 11% matched to three mosquito species belonging to Culicomorpha (*Cu. quinquefasciatus*, *Ae. aegypti* and *An. gambiae*). About 79.47 and 81% of the unigenes in *Bactrocera dorsalis* and *Glossina morsitans morsitans*, were previously reported to be closely related to *Drosophila*, respectively [22], [23]. In contrast to Bibionomorpha (gall midge), Ephydroidea (e.g., *Drosophila*) are closer to Tephritoidea (e.g., *B. dorsalis, G. morsitans*). However, the closest relatives are the lower dipterans, the Culicomorpha (mosquito) and Bibionomorpha [24]. Our finding that 43.18% of the unigenes was most closely related to mosquito was consistent with these previous results. Thus, the transcriptome analysis more accurately reflects the evolutionary process.

Thus far, only 39 nucleotide sequences and 1,219 expressed sequence tags of OWBM are available in GenBank (prior to January 10, 2013). Our transcriptome and gene expression profiling data greatly enriches the current OWBM database and will be resourceful to the identification of novel genes, chemical targets, and developmental mechanisms, especially the diapause mechanisms of OWBM.

Diapause is divided into six phases, namely induction, preparation, initiation, maintenance, termination and quiescence, according to the definition proposed by Kostal [14]. It is characterized by reduced metabolic rate, simultaneous activation of associated metabolic pathways and gene expression of metabolic enzymes. Larval diapause is usually characterized by a shut down of the brain-prothoracic gland axis and the prothoracic gland (PG) fails to synthesize the ecdysteroids. This may be caused by either the failure of the brain to release the neuropeptide prothoracicotropic hormone (PTTH) needed to stimulate the PG [25] or by failure of the PG to respond to PTTH until an adequate period of chilling has been experienced [26]. Specific patterns of gene expression and unique metabolic profiles characterize diapause and the cross-talk between fat body and brain regulates developmental arrest [27]. So the alternative life-histories of OWBM, diapause vs non-diapause, are associated with clear gene abundance differences. Our results showed that among 3,005 genes with significant differential expression levels between NDOL and DOL, 1,893 were up-regulated and 1,112 genes were down-regulated in NDOL compared to DOL.

### HSPs

As molecular chaperones, heat shock proteins are involved in maintaining the integrity of key metabolic enzymes or structural proteins at low temperatures during winter or high temperatures during summer [28]. Moreover, heat shock proteins may contribute directly to the regulation of diapause by assuring a cell cycle arrest, which has been linked to expression of both small heat shock proteins [29] and Hsp70 [30], [31].

Small heat shock proteins (sHsps) are a superfamily of proteins that contain an α-crystalline domain with molecular weights ranging from 12–43 kDa depending on variable N- and C-terminal extensions.

sHsps contribute to thermal resistance [32], [33] and their expression patterns change when insects enter into diapause [34]–[36]. In the present study, the expression levels of sHsps were not significantly different between NDOL and DOL. This showed that these sHsps are not associated with diapause.

Hsps are up-regulated during diapause to increase tolerance to environmental stresses such as temperature and are down-regulated when more favorable conditions return. Hsps expression during DOL (diapause stage) indicates a typical stress response when all Hsps have high expression levels including members of Hsp70 and Hsp90 families. In contrast, the gene expression of a cognate of Hsp70, Hsc70, remains unchanged. Perhaps OWBM receives cues to reinitiate development (having just terminated diapause) when the HSPs expression declines to normal levels. In the blow fly *Lucilia sericata*, the expressions of *Hsp70* and *Hsp90* are not regulated in response to diapause, but *Hsp90* transcripts were promptly up-regulated after termination of diapause and during post-diapause development. In the flesh fly *Sarcophaga crassipalpis*, Hsp70 family and at least four members of the sHsp are highly up-regulated in diapausing pupae without temperature stress, and levels of the transcripts decline when diapause is terminated and post-diapause development followed [37], [38]. Up-regulation of Hsps during diapause may be a major factor contributing to cold-hardiness of overwintering insects [38]. In contrast, transcripts of *Hsp90* in *S. crassipalpis* show a pattern different from *Hsp70*: they are down-regulated in diapausing pupae, but expression is restored after diapause is terminated and post-diapause development is allowed [39]. This suggests distinct roles for the different heat shock proteins during diapause.

### Metabolic Enzyme Gene Expression

Utilization of sorbitol is controlled by NAD-sorbitol dehydrogenase (SDH). In OWBM, SDH2a and SDH2b were significantly lower in NDOL than DOL. Diapause-related changes in SDH expression is also observed in other insects. In *Bombyx mori,* SDH is expressed late during embryonic diapause [40], [41] and declines during the termination of diapause in the adult heteropteran bug, *Pyrrhocoris apterus* [42]. In *Wyeomyia smithii*, SDH-1 and SDH-2 are highly expressed during larval diapause, and are down-regulated after termination of diapause [43]. High levels of SDH expression during diapause and declining levels at termination could be related to recovery from poor oxygen conditions and a release from the need for cryoprotection [43] . It is yet to be determined whether such changes could contribute to the termination process.

Activity of SDH in OWBM larvae was not detectable in wheat crop that mature in November although it increased sharply from January to April [44]. Changing patterns in the amount of OWBM transcripts could not be correlated to the activity of SDH. The underlying enzymatic differences between diapause and non-diapause, however, may not be only due to differences in gene expression but could also be attributed to the presence or absence of substrates/cofactors/inhibitors. Real-time PCR in different development stages, showed that SDH expression level was higher only in OWBM adults although in *Drosophila*, SDH transcripts were present in larvae and adults and a low expression was detected in pupae [45].

Trehalose, the major insect blood sugar, has previously been considered a cyroprotectant in some insect species [46]–[48]. During diapause, trehalose content was maintained at a high level but decreased after diapause termination. [49]. Alternately, trehalase activity was lower but increased after diapause termination. In different diapausing stages of OWBM larvae a correlation has been reported between high trehalase activity and energy transition [50]. Two unigenes encoding proteins with trehalase activity (TREH1 and TREH2) had different expression patterns. The expression of TREH1 in NDOL was up-regulated and TREH2 in NDOL was down-regulated when compared to DOL. This indicated that one TREH may have real role participating in the metabolism of trehalose.

Glycogen phosphorylase (GP) is more likely to be activated and provide glucose substrates for glycerol synthesis at low temperatures, providing direct environmental control of metabolic flux via protein phosphorylation reactions [51]. The relative levels of transcripts of genes coding for GP was significantly lower in NDOL than DOL. This result was consistent with a decrease in GP activity after diapause termination [50].

The activity of FBPA increased after diapause termination and was considered to form glycogen from trehalose to supply energy for development [52]. But the FBPA was down-regulated in NDOL than DOL and the FBPA transcript levels reached a peak in OWBM adults. In *Helicoverpa armigera*, FBPA transcripts function in metabolism and energy [53].

TCA cycle may be a checkpoint of diapause for regulating different forms of animal dormancy. In *H. armigera*, leves of isocitrate are higher in the hemolymph of diapausing pupae, whereas levels of pyruvate aer lower in diapausing papae. IDH, which participate in the TCA cycle, was up-regulated in NDOL. This result implies that the up-regulated IDH may incite the TCA cycle in NDOL. In diapausing pupae of the flesh fly, *S. crassipalpis*, the TCA cycle is suppressed and the metabolic intermediates from the TCA cycle are also reduced [54]. In *H. armigera*, diapausing entrance is accompanied by a decrease in the release of intermediates from the fat body, resulting in a significant reduction of TCA activity in the brain. The levels of glucose, pyruvate are lower in diapausing pupae, whereas levels of isocitrate, trehalose are higher in diapausing pupae [27], [53]. The results are consistent with our studies. In NDOL of OWBM, pyruvate dehydrogenase (PD) and ATP-citrate synthase were down-regulated. These differences in metabolic profiles of diapausing and nondiapausing larvae of OWBM suggest that the possibility that this information may be used as a component of the signaling system that regulates diapause. And further studies are required to verify.

Juvenile hormone epoxide hydrolase (JHEHs) and juvenile hormone esterase (JHE) were down-regulated in NDOL than DOL. JHEHs are a family of enzymes that hydrolyze juvenile hormones (JHs) and JHE are important for the onset of metamorphosis which is demonstrated in a variety of insect species [55]. JHIII titre in over-wintering OWBM larvae was higher than over-autumning larvae [56]. But in *Laspeyresia pomonella*, diapause was initiated by a relatively high titer of JH during the last larval instar [57]. In addition, diapausing *Sesamia nonagrioides* larvae maintained high JH titer and JHE activity. Real-time PCR results showed that the expression of JHEH decreased in pre-pupae. Relative to the cocoon, the expression level of JHE was significantly higher in pre-larvae and reduced to a lower level in early-pupae. These were two different expression patterns with different roles in JH clearance and conversion to acid. These results indicated that JHE was crucial for the switch between larvae and pupae in OWBM.

In conclusion, we sequenced the transcriptome of OWBM and constructed a DGE library. These efforts revealed a large number of genes, both of known and unknown functions, greatly expanding the amount of genetic information available for this species and providing a profile of its diapause and non-diapause stages. The results of this study would lead to better understanding of the molecular mechanism underlying diapause in OWBM.

## Supporting Information

File S1 **Table S1-Table S9, Figure S1–S3.** Table S1 Top blast hits from NCBI nr database Table S2 GO annotation of unigenes Table S3 KO annotation of unigenes Table S4 Genes expressed differentially between NDOL and DOL Table S5 Top ten differentially expressed genes Table S6 GO function and KEGG Pathway analysis results of NDOL vs DOL Talbe S7 Hsp genes expressed differentially between NDOL and DOL Table S8 Verification of differentially expressed genes by qRT-PCR Table S9 Real time PCR primers Figure S1. Classification of raw reads, distribution of distinct clean tags and genes’ coverage in NDOL and DOL. A, Percentages of adaptors, containing N, low quality and clean reads. Numbers in parentheses indicate the percentage of each type of tag among the total raw tags. B, Numbers indicate the range of copy numbers for a specific category of reads. The data in parentheses indicate the percentage of corresponding reads among the total distinct tags. C, Percentage of a gene covered by reads. Figure S2 Sequencing Saturation Analysis Figure S3 Phylogenetic analysis of SDH genes.
(ZIP)
