# Supplementary figures and images for: Global Transcriptome Analysis of Orange Wheat Blossom Midge, Sitodiplosis mosellana (Gehin) (Diptera: Cecidomyiidae) to Identify Candidate Transcripts Regulating Diapause
Source: PLoS One. 2013 Aug 5;8(8):e71564. doi: 10.1371/journal.pone.0071564 (PMC3733836; doi:10.1371/journal.pone.0071564)

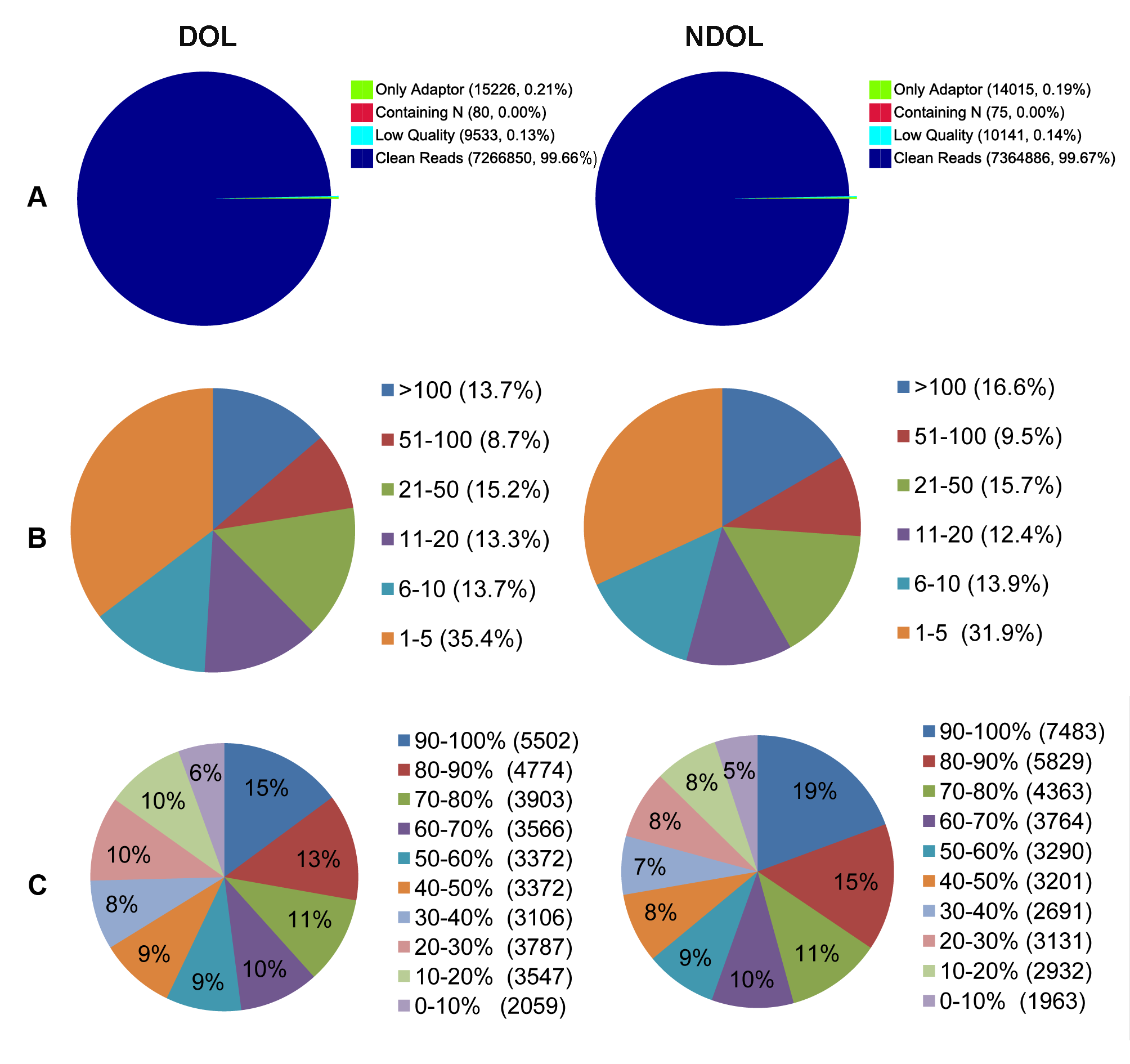

Supplement: File S1 — Table S1-Table S9, Figure S1–S3. Table S1 Top blast hits from NCBI nr database Table S2 GO annotation of unigenes Table S3 KO annotation of unigenes Table S4 Genes expressed differentially between NDOL and DOL Table S5 Top ten differentially expressed genes Table S6 GO function and KEGG Pathway analysis results of NDOL vs DOL Talbe S7 Hsp genes expressed differentially between NDOL and DOL Table S8 Verification of differentially expressed genes by qRT-PCR Table S9 Real time PCR primers Figure S1. Classification of raw reads, distribution of distinct clean tags and genes’ coverage in NDOL and DOL. A, Percentages of adaptors, containing N, low quality and clean reads. Numbers in parentheses indicate the percentage of each type of tag among the total raw tags. B, Numbers indicate the range of copy numbers for a specific category of reads. The data in parentheses indicate the percentage of corresponding reads among the total distinct tags. C, Percentage of a gene covered by reads. Figure S2 Sequencing Saturation Analysis Figure S3 Phylogenetic analysis of SDH genes. (ZIP) [file pone.0071564.s001.zip › Supporting Information/Supporting Information/Figure S1.tif]

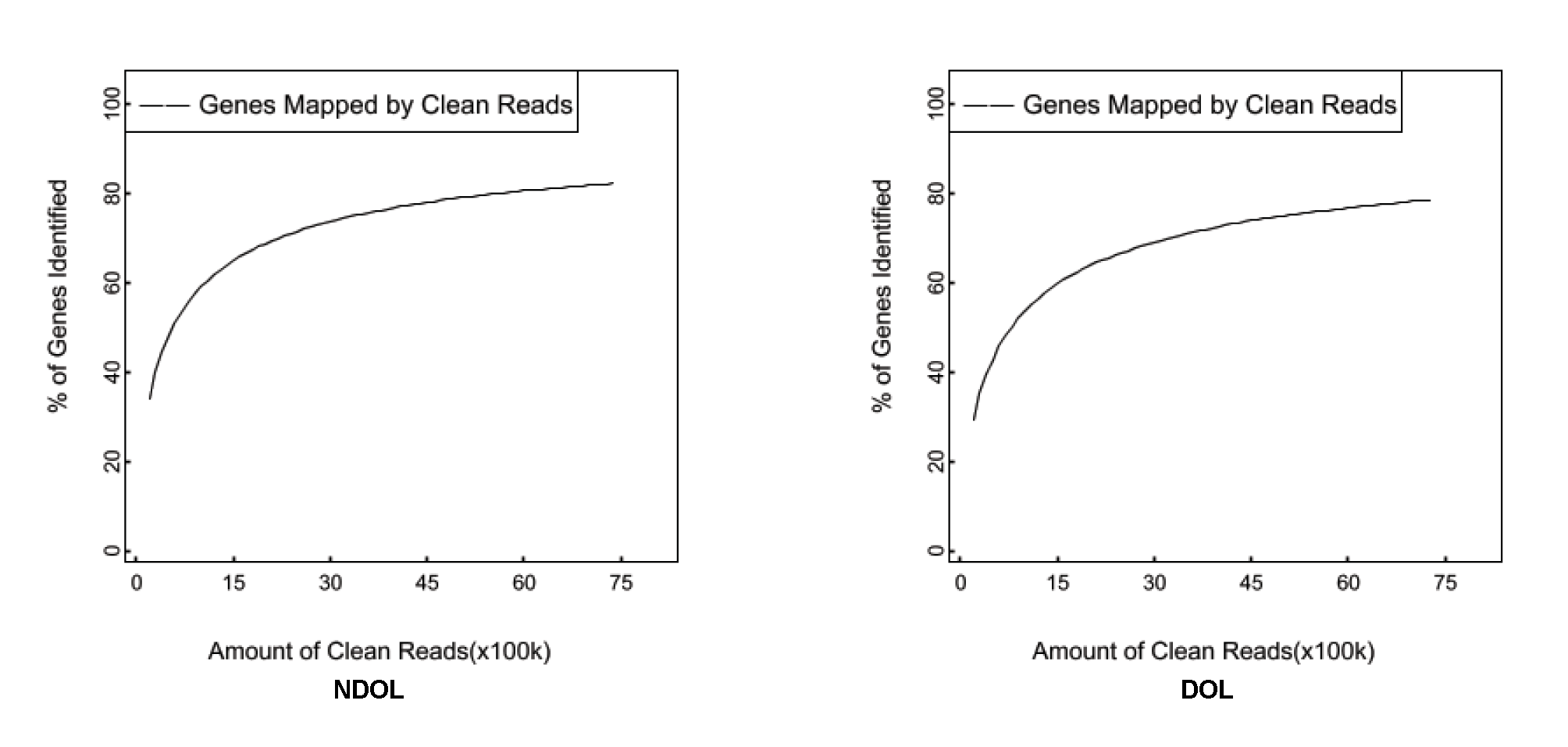

Supplement: File S1 — Table S1-Table S9, Figure S1–S3. Table S1 Top blast hits from NCBI nr database Table S2 GO annotation of unigenes Table S3 KO annotation of unigenes Table S4 Genes expressed differentially between NDOL and DOL Table S5 Top ten differentially expressed genes Table S6 GO function and KEGG Pathway analysis results of NDOL vs DOL Talbe S7 Hsp genes expressed differentially between NDOL and DOL Table S8 Verification of differentially expressed genes by qRT-PCR Table S9 Real time PCR primers Figure S1. Classification of raw reads, distribution of distinct clean tags and genes’ coverage in NDOL and DOL. A, Percentages of adaptors, containing N, low quality and clean reads. Numbers in parentheses indicate the percentage of each type of tag among the total raw tags. B, Numbers indicate the range of copy numbers for a specific category of reads. The data in parentheses indicate the percentage of corresponding reads among the total distinct tags. C, Percentage of a gene covered by reads. Figure S2 Sequencing Saturation Analysis Figure S3 Phylogenetic analysis of SDH genes. (ZIP) [file pone.0071564.s001.zip › Supporting Information/Supporting Information/Figure S2.tif]

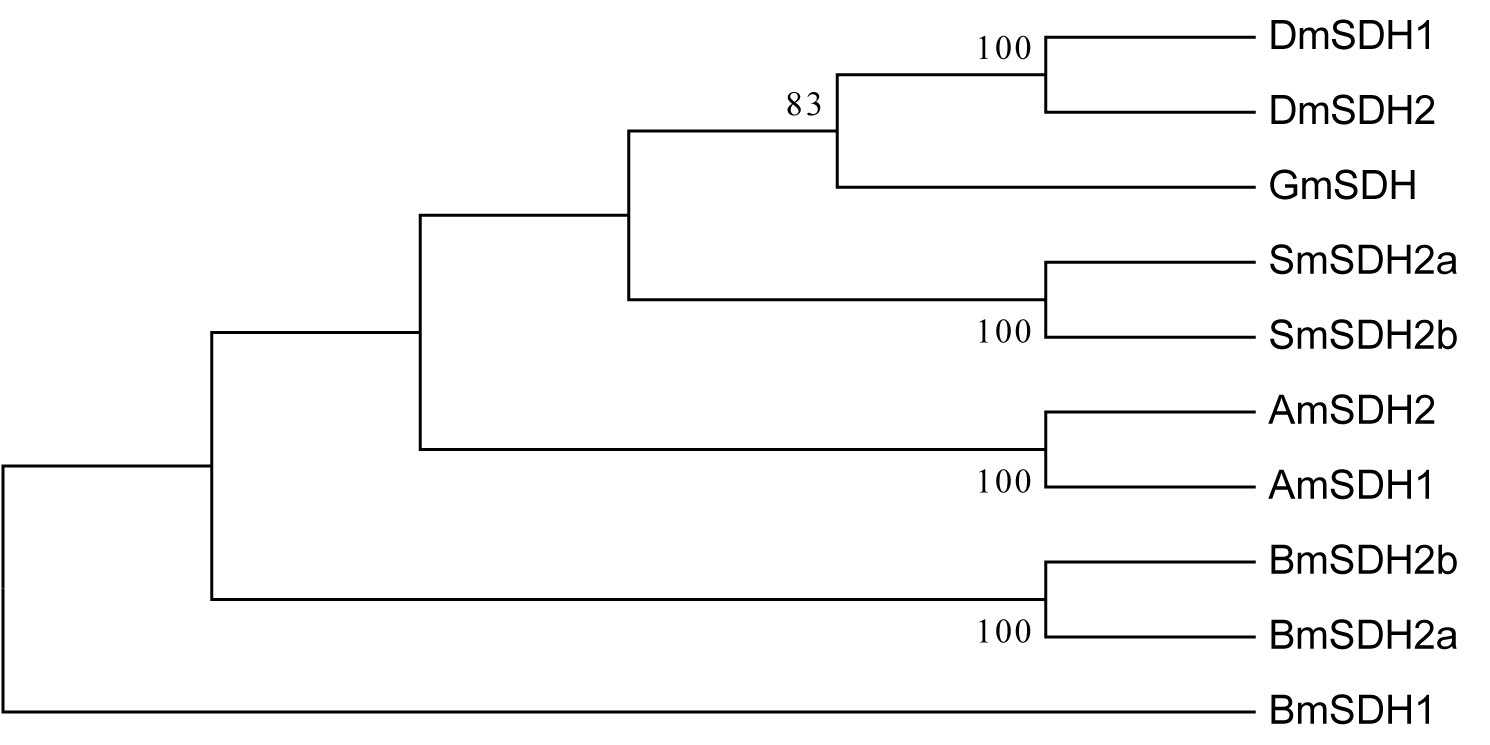

Supplement: File S1 — Table S1-Table S9, Figure S1–S3. Table S1 Top blast hits from NCBI nr database Table S2 GO annotation of unigenes Table S3 KO annotation of unigenes Table S4 Genes expressed differentially between NDOL and DOL Table S5 Top ten differentially expressed genes Table S6 GO function and KEGG Pathway analysis results of NDOL vs DOL Talbe S7 Hsp genes expressed differentially between NDOL and DOL Table S8 Verification of differentially expressed genes by qRT-PCR Table S9 Real time PCR primers Figure S1. Classification of raw reads, distribution of distinct clean tags and genes’ coverage in NDOL and DOL. A, Percentages of adaptors, containing N, low quality and clean reads. Numbers in parentheses indicate the percentage of each type of tag among the total raw tags. B, Numbers indicate the range of copy numbers for a specific category of reads. The data in parentheses indicate the percentage of corresponding reads among the total distinct tags. C, Percentage of a gene covered by reads. Figure S2 Sequencing Saturation Analysis Figure S3 Phylogenetic analysis of SDH genes. (ZIP) [file pone.0071564.s001.zip › Supporting Information/Supporting Information/Figure S3.tif]
